# Challenges, salutogenic resources and adolescent agency: a qualitative study in a public school in Peru

**DOI:** 10.3389/fpubh.2026.1824105

**Published:** 2026-04-29

**Authors:** Maricela M. Guevara-Cabello, Bily Vargas Huamancayo, Dolors Juvinyà-Canal, Glòria Reig-Garcia

**Affiliations:** 1 Doctoral Program in Molecular Biology, Biomedicine and Health, Universitat de Girona, Girona, Catalonia, Spain; 2 Faculty of Science and Engineering, School of Nutrition, Universidad Peruana Cayetano Heredia, Lima, Peru; 3 Nursing Department, Research Group on Health and Healthcare, University of Girona, Girona, Catalonia, Spain

**Keywords:** adolescence, agency, daily challenges, general resistance resources, health promotion, salutogenesis

## Abstract

**Introduction:**

Adolescents living in socioeconomically vulnerable environments face multiple interrelated academic, emotional, relational, and economic challenges that shape their well-being and development. Research has largely prioritised risk-based and adult-centred perspectives, often overlooking adolescents’ capacities and strengths. This study explored how adolescents in a Peruvian public school perceive daily challenges, mobilise General Resistance Resources (GRRs), and exercise agency from a salutogenic perspective.

**Methods:**

A qualitative study was conducted in a public secondary school in Acobamba, Peru. Twenty-eight adolescents (21 females and 7 males) and 6 teachers participated. Data were collected through month-long personal diaries, adolescent focus groups, and semi-structured interviews with teachers. A reflective thematic analysis was applied with equal rigour to adolescent and teacher data, enabling comparative analysis. Coding was inductive and supported by ATLAS.ti, integrating gender and salutogenesis as analytical lenses.

**Results:**

Adolescents identified academic, relational, economic, family, and everyday challenges. They mobilised diverse GRRs (personal, social, spiritual, digital, and institutional) for emotional regulation, problem-solving, and meaning-making. Agency emerged as a dynamic context-dependent capacity shaped by the perceived impact of challenges and available resources, consistent with bounded agency. Gender differences were observed in the recognition of challenges, resources, and agency. Teachers recognised some resources and emerging agency in adolescents however emphasised risk, dependency, and limited autonomy, contrasting with adolescents’ more active accounts.

**Conclusion:**

Adolescent well-being reflects the interaction between structural conditions, relational contexts, and resource mobilisation. Resources must be recognised, activated, and socially validated within a constrained agency. Bridging the gap between adolescent and adult perspectives is essential. Health promotion should strengthen adolescent capacities, address structural barriers, and incorporate gender and contextual perspectives.

## 1 Introduction

Adolescence is a crucial stage of human development, characterised by diverse and profound changes that shape the construction of identity, progressive autonomy and the acquisition of life skills (1, 2). In contexts of socio-economic vulnerability, these changes occur in settings marked by structural inequalities that directly influence adolescent well-being and comprehensive development. Globally, adolescents represent a significant proportion of the population, and in Peru they constitute approximately 10% of the national total (3), Huancavelica being one of the regions with a particularly large youth population base, and currently maintaining significant levels of poverty and inequality (4).

In this context, adolescents face multiple challenges such as adolescent pregnancy, especially in rural areas and in lower income quintiles (5, 6) as well as important indicators of morbidity and mortality in regions such as Huancavelica (7). In relation to mental health, there has been an increase in suicide attempts and episodes of depression, with women being the most affected and the age range being 15 to 19 years old (8). Furthermore, domestic and school violence continues to be a significant problem, including physical, psychological and sexual violence in school environments (9, 10). Educational gaps, school dropout rates and regional inequalities specific to Huancavelica exacerbate this situation (11).

In response, the Peruvian government has implemented various regulations and sectoral strategies aimed at providing comprehensive care for adolescents, such as national standards for comprehensive care, educational guidelines, and national multisectoral plans (12–16). However, these initiatives have a predominantly deficit or disease and/or risk-centred approach, mainly being activated when the problem has already arisen. This approach, which is often adult-centred, tends to place adolescents in a passive role in relation to their contexts (3).

On the other hand, the salutogenic model proposed by Antonovsky offers a perspective focused on the processes that generate and sustain health (2). Under this theory, well-being is explained by the mobilisation of General Resistance Resources (GRR) and the development of a Sense of Coherence (SOC) (17), understood as the ability to perceive life as understandable, manageable, and meaningful (18). Salutogenic framework includes individual, relational, and structural resources that enable individuals to cope with stressors, while high SOC is associated with better mental health indicators, lower adoption of risk behaviours, and greater overall well-being in adolescence, contributing to their comprehensive development (19–21).

Factors such as gender, socioeconomic status, and family composition influence the development of SOC (22, 23), which suggests that its strengthening depends on broader ecological conditions.

However, the availability of resources does not guarantee their activation. Evidence from salutogenic research and health promotion suggests that resources only contribute to well-being when people are able to recognise them, give them meaning, and actively mobilise them in line with their own goals and context (24, 25). In this context, the salutogenic model emphasises the availability and management of resources and the construction of meaning; however, as Antonovsky (59) points out, it does not fully address people’s capacity to act upon these resources in the pursuit of meaningful goals. Therefore, incorporating autonomy and the capacity for action into this framework allows for a better understanding of how people not only cope with stressors, but also actively use resources to shape their life trajectories (26). It is in this context the concept of conditional agency emerges, defined as people’s capacity for action, which is conditioned and limited by structural, social and institutional factors, as well as by the resources available in their environment (27). From a public health perspective, analysing how adolescents exercise agency in structurally vulnerable contexts allows us to transcend models focused exclusively on risk, as well as to position adolescents in a more active role, capable of understanding their contexts and making decisions, moving towards a more holistic and dynamic understanding of adolescent well-being.

International research has examined adolescent agency through qualitative and comparative approaches in contexts of vulnerability, particularly in the Global South, demonstrating that it is not only conceptualised but also assessed on the basis of adolescents’ ability to recognise, mobilise and negotiate resources in their daily lives. Qualitative studies have identified agency through coping strategies, decision-making and the use of support networks, demonstrating how adolescents actively manage situations of adversity (28). Furthermore, comparative research has shown that agency manifests itself in different ways across countries, depending on the context, and is influenced by structural, cultural and gender-related factors (29). In this line, approaches have been developed to measure agency in adolescents based on dimensions such as voice, decision-making and control over their actions, highlighting the need for contextual adaptations to ensure their proper assessment (30). In contexts of high vulnerability, agency has been understood as a relational and situated process, in which adolescents develop everyday strategies to cope with violence, stress and uncertainty, actively negotiating their well-being, such us such as hypervigilance, avoidance and emotional regulation, within environments marked by structural inequalities and institutional constraints (31). Overall, this evidence suggests that agency should be understood as a dynamic process that emerges from the interaction between available resources and environmental constraints, reinforcing the importance of integrating it into frameworks such as salutogenesis.

Despite growing interest in adolescent health, there is still limited evidence that examines, from a salutogenic approach and in rural contexts of high social vulnerability such us acobamba - Perú, how adolescents identify their daily challenges, mobilise general resources of resistance, and exercise agency within school and community settings. In this context, the present study seeks to provide contextualised evidence by analysing how challenges, resources, and agency are articulated in the daily experience of adolescents.

## 2 Materials and methods

### 2.1 Study design

A qualitative study was conducted, adopting a phenomenological and socio-constructivist approach aimed at understanding how adolescents interpret their daily experiences in relation to the challenges they face. This approach made it possible to analyse how these experiences are linked to the mobilisation of resources and processes of agency in their daily lives, placing their narratives within the social context in which they develop (32).

### 2.2 Setting and participants

The study was conducted in a public secondary school located in the province of Acobamba, Huancavelica region, Peru, characterised by high levels of socio-economic vulnerability and structural gaps.

The population consisted of adolescents enrolled in the third year of secondary education during the study period. The entire group (*N* = 42) was invited to participate, in order to avoid any potential exclusions within the classroom that could cause discomfort among adolescents; this decision was coordinated with the school authorities and the teaching staff.

Participation was voluntary and required the informed consent of adolescents and the informed consent of their parents or guardians. Of the 42 students invited, 28 agreed to participate and completed the personal diaries, while 14 decided not to participate after receiving information about the study. No students were excluded by the research team. This voluntary participation may have contributed to the observed gender imbalance in the sample.

As the students were in the same year group and class, no significant demographic differences were identified between those who participated and those who did not.

Regarding the teachers, six professionals were invited to participate, selected through purposive sampling based on their role within the year group under study. The group included the tutoring coordinator, who was responsible for managing the school’s form tutor programme and training other teachers to work with adolescents, as well as the tutors and teaching assistants assigned to the third year of secondary school, who worked directly with the students. This selection enabled us to gather perspectives directly linked to the participating group, in order to incorporate the adult perspective on adolescent experiences and development processes in the school context.

The sampling method chosen could lead to potential biases. The voluntary participation of students could imply that those who agreed to take part are more inclined towards reflection or engagement in this type of activity. Furthermore, the intentional selection of teachers, based on their direct link to the school year, may have favoured perspectives closer to tutorial support and knowledge of the students, leaving out other viewpoints within the institution. Both aspects could influence the nature of the findings, particularly in the identification and description of resources and experiences, as well as in teachers’ perceptions of adolescence; therefore, the results should be interpreted with these potential limitations in mind.

### 2.3 Data collection procedures

Data collection was carried out between September and December 2025, using three qualitative techniques.

*Personal diaries for adolescents*: A semi-structured journal was designed with three thematic blocks: (I) Who am I and what is my context?, with questions related to exploring identity and the environment in which they live; (II) My day-to-day life, focused on everyday experiences, challenges and emotions; and (III) Weekly reflection, aimed at complementing the discussions raised in their day-to-day lives and promoting processes of personal meaning and analysis. For 1 month, the adolescents anonymously recorded their experiences, perceived challenges, resources used, and agency. This technique provided access to personal narratives and reflective processes in people’s natural context, encouraging reflection, the development of different skills and metacognition (33).

Prior to its implementation, the instrument was validated with a volunteer group of adolescents to ensure conceptual clarity and cultural appropriateness. Based on their suggestions, adjustments were made to the visual design, incorporating a visual guide that provided guidance on the frequency and method of recording, as well as adjustments to the format of the diary, without interfering with the content.

No minimum number of entries was established, as the aim was to respect the voluntary nature of the process. The diaries were handed out at school and completed at home. Guardians reinforced procedural compliance without interfering with the content of the diary. Once collected, the diaries were scanned and uploaded directly into ATLAS.ti software (RRID:SCR_014836) for analysis.

Although each diary allowed for up to 168 possible entries, the volume of entries across the 28 diaries reflected the inherent variability of a naturalistic design. While some participants maintained a high level of consistency, others recorded entries more intermittently, particularly in the daily entries. This variability was not considered a limitation, but rather reflected the subjective relevance of the experiences.

To ensure the robustness of the analysis, aspects less developed in the diaries were explored in greater depth through focus groups. This provided sufficient analytical depth and allowed saturation to be reached.

*Focus groups*: Two focus groups were held, differentiated by gender, each lasting approximately 120 min. The objective was to explore and reflect collectively on the preliminary findings from the diaries. This technique allowed perceptions, meanings and experiences to be explored collectively through interaction between participants, which diversifies and enriches the responses (34, 35). The objective was to delve deeper and reflect collectively on the preliminary findings from the diaries.

Participatory and arts-based methodologies were used to encourage creative expression and the shared construction of meaning (36). The sessions were recorded with prior consent and transcribed verbatim.

*Semi-structured interviews with teachers*, aimed at gathering adult representations of adolescence. This technique allowed perceptions and experiences to be explored from the participants’ perspective, maintaining a balance between the flexibility of the conversation and the thematic orientation of the research, while maintaining objectivity and reliability (37), as well as complementarity and triangulation of data (38). The questions addressed work experience with adolescents, perceptions of challenges and resources, school dynamics, and adolescent agency processes.

The interviews were recorded with prior informed consent and transcribed verbatim for subsequent analysis.

### 2.4 Data analysis

The analysis was conducted using a reflective thematic analysis approach, a qualitative approach used to identify,analyse and organise patterns of meaning within a data set, relevant for exploring experiences, perceptions and social constructions (39, 40). The analysis process included repeated readings of the diaries and transcripts of the focus groups and interviews, following the phases proposed by Braun and Clarke (60): familiarisation with the data, generation of initial codes, construction of themes, review of themes, definition and naming of themes, and preparation of the final report. The gender perspective guided the entire analytical process. The process was iterative and involved a constant movement between the data, codes and themes. ATLAS.ti software (v.23) was used to facilitate the organisation, coding and traceability of the analytical process.

#### 2.4.1 Coding and theme development

The coding was carried out inductively, starting from the verbatim data and moving towards progressively higher levels of conceptual abstraction. This process involved generating initial codes, grouping them into sub-themes and, subsequently, constructing themes.

For example, the initial code “Difficulty concentrating/understanding the lesson” was grouped under the sub-theme “Problems with understanding academic subjects,” which contributed to the construction of the theme “Academic challenges.” Similarly, expressions such as ‘Support in God in difficult situations’ were coded and grouped under the sub-theme ‘Spirituality as a resource’, forming part of the theme “Personal resources.”

This process was carried out iteratively, allowing for continuous refinement of the thematic structure. A sample of the relationship between codes, sub-themes and themes was systematised in an analytical matrix (Supplementary Table 1), in order to ensure transparency in the construction of the findings.

#### 2.4.2 Triangulation and analytical consistency

To strengthen the credibility of the analysis, the principal investigator conducted the analysis in accordance with the ethical approval obtained for the study, which established that access to identifiable data would be restricted to a single researcher to ensure confidentiality and data protection. Subsequently, the coding framework and emerging themes were discussed with other members of the research team in consensus meetings. During these sessions, interpretations were compared and discrepancies were discussed and resolved through reasoned agreement, enabling the refinement of the coding system and the final thematic structure.

Reflective thematic analysis was applied with the same rigour and systematic approach to both the adolescents’ data (diaries and focus groups) and the teacher interviews. The teacher interviews were not used merely as contextual background, but as an independent data set that was coded inductively. This allowed for an explicit comparative analysis to identify whether the adults’ discourses converged with, enriched or contradicted the adolescents’ experiences, ultimately integrating both perspectives into the construction of the overarching themes.

To strengthen the confirmability of the findings, a triangulation of sources and methods was carried out by cross-analysing personal diaries, focus groups and interviews with teachers. This process enabled the identification of relevant convergences and divergences: While the focus groups expressed challenges in general terms (e.g., “difficulties with studying or in the school”), the personal diaries provided more depth, highlighting specific difficulties such as problems with concentration and comprehension in class or in specific topic. Meanwhile, the interviews with teachers allowed these perceptions to be cross-checked and validated, with agreement on the academic difficulties but differences in the interpretation of adolescence. This triangulation enabled the phenomenon to be captured from subjective, collective and institutional perspectives, strengthening the interpretative robustness of the analysis.

#### 2.4.3 Reflexivity and the research team’s position

The research team adopted a position of critical reflexivity throughout the analytical process. It is acknowledged that their training and experience in the field of health promotion and salutogenesis may have influenced their sensitivity towards identifying health resources and assets.

To achieve this, a professional with a clinical-care orientation was incorporated into the research team; her perspective, centred on understanding needs, risks and health conditions, complemented the predominant focus on health promotion and salutogenesis. This dialogue between approaches allowed for a broader interpretative framework, avoiding the limitation of the analysis to a single perspective and reducing the risk of confirmation bias. Furthermore, strategies such as keeping field diaries and holding reflective discussions between the two approaches, fostered a richer and more nuanced interpretation of the data, integrating both resources and challenges within the adolescents’ experiences. This process helped to strengthen the study’s confirmability, ensuring that the findings emerged from the data rather than from the research team’s prior assumptions or predefined theoretical categories.

Furthermore, the composition of the team, with members based in Spain and Peru, provided a complementary perspective: on the one hand, a closer understanding of the participants’ socio-economic and rural context; and on the other, an analytical distance that facilitated the identification of broader patterns relating to the phenomenon under study. Particular attention was paid to the adult-centred tensions identified in the findings. As a team composed of adult female researchers, we sought to avoid paternalistic interpretations and potential gender biases. Through ongoing reflective discussions, we questioned normative assumptions about adolescence, favouring an interpretation that prioritised the voices and experiences of the participants.

#### 2.4.4 Data saturation

Data collection and analysis were carried out simultaneously. Conceptual saturation was deemed to have been reached when the final data sources did not yield new codes or alter the existing thematic structure, but rather confirmed previously identified patterns. At this point, the inclusion of further data was deemed redundant for understanding the phenomenon under study.

#### 2.4.5 Compliance with quality criteria

The analytical process and its reporting were structured in accordance with the items of the COREQ guide for qualitative research (41), with the aim of ensuring transparency, methodological rigour and consistency in qualitative research.

### 2.5 Research team and quality standards (COREQ)

Data collection and analysis were carried out by the principal investigator, a PhD student in the field of Health Promotion and Health Services, with specialist training in health promotion and qualitative methodology. Her approach was grounded in a salutogenic perspective, focusing on a positive view of health and adolescence, which was taken into account throughout the analytical process. There were no prior relationships between the research team and the participants (students or teachers), which facilitated interaction free from pre-existing connections.

The interviews and focus groups were conducted in private rooms within the school, ensuring confidentiality, freedom from interruptions and an environment conducive to free expression. Furthermore, the conditions were characterised by an atmosphere of trust and openness, facilitated by the prior use of personal diaries, which were handed out to be completed in the spaces the adolescents themselves deemed most appropriate, respecting their privacy and the nature of their everyday lives. This allowed participants to reflect on their experiences prior to the group sessions. It was emphasised that participation was voluntary and had no bearing on their assessment or academic performance within the school environment.

Finally, data saturation was determined through a concurrent analysis process, according to the point 2.4.

### 2.6 Data visualisation and categorisation

To facilitate the interpretation of the findings, treemaps were created (Supplementary Figures 1–3). These figures represent the structure and relative distribution of the categories and subcategories that emerged from the thematic analysis. The size of the areas in the graphs is based on the relative frequency of the coded segments associated with each category, identified during the coding process in the ATLAS.ti software.

It is important to note that, given the qualitative nature of the study, these frequencies are used exclusively for illustrative purposes, with the aim of showing the thematic density and relevance of certain challenges and resources within the participants’ narratives, and not to draw statistical inferences.

### 2.7 Ethical considerations

This study was reviewed and approved by the Institutional Research Ethics Committee (CIEI) of Cayetano Heredia Peruvian University (CIEI-E-291-36-25). Written informed consent was obtained from the parents or legal guardians of all participating adolescents, and assent was obtained from the adolescents prior to participation. Participants were informed about the aims of the study, the voluntary nature of participation, and their right to withdraw at any time. All data were anonymized and handled confidentially.

## 3 Results

Twenty-eight adolescents (21 females and 7 males) aged between 13 and 16 years old, enrolled in the third year of secondary education, participated, as well as six teachers (three females and three males) with tutoring and student support roles. In terms of the characteristics of the adolescents, most reported living with both parents, although single-parent families, mainly maternal, and living with extended family were also identified. The main activity was studying, although some adolescents combined study and work. Most identified as single, with some romantic experiences, and Catholic religious affiliation predominated, followed by Evangelical.

Reflective thematic analysis identified three major themes, organised into several subthemes: (1) daily challenges in a context of structural vulnerability, including academic, emotional and relational, economic, everyday and family challenges; (2) general resistance resources, referring to personal, family, social, digital, institutional and community resources mobilised by adolescents; and (3) conditioned agency and coping strategies, which describes adolescents’ perceived capacity to act and the strategies they use to face everyday challenges. Teachers’ perspectives were integrated across themes to contrast and contextualise adolescents’ narratives, enabling a dialectical analysis between the two groups.

### 3.1 Daily challenges in a context of structural vulnerability

Adolescents described their daily lives as experiences marked by multiple learning opportunities, as well as challenges that arise and interact at different levels: academic, emotional, relational, economic, and everyday. Supplementary Figure 1 shows the distribution of the main challenges reported by adolescents. However, the comparative analysis reveals a tension in interpretation: while adolescents describe these experiences from their own lived perspective, teachers tend to interpret them within broader frameworks associated with risk, a breakdown of boundaries and vulnerability.

#### 3.1.1 Academic challenges

In the academic sphere, situations of school pressure, difficulties in understanding academic content, conflicts with teachers, experiences of bullying and discrimination among peers emerged. Some accounts revealed episodes of verbal violence or social exclusion in the classroom, which generate stress, frustration and demotivation at school.

One adolescent pointed out:

*‘Maths lessons were difficult and boring[…] the teachers did not give us time, it was tiring’ (Adolescent, female)*.

Likewise, experiences of normalised discrimination based on skin colour and physical appearance within the school context emerged.

*‘Discrimination […] we think it’s just a game […] but it hurts us*.*’ (Adolescent, female)*.

Situations of direct violence were also reported.

*‘A classmate was hitting all the girls’ (Adolescent, female)*.

Female adolescents tended to verbalise their challenging academic experiences in greater detail, while male adolescents did not explicitly identify them or attributed them to external factors, such as lack of internet access or dislike of certain subjects.

On the other hand, teachers’ discourse does not focus on academic difficulty in itself, but tends to reinterpret these situations as manifestations of problematic behaviour or a loss of authority typical of adolescence:

*‘Students are already quite empowered, now they are in control of the situation’ (Teacher, female)*.

Some teachers interpret so-called adolescent “empowerment” as excessive autonomy or a lack of control, associated with behaviours considered inappropriate, such as “doing what they want” or a loss of respect for adult authority. In this context, certain institutions that promote discourses on rights or personal development are perceived as generating disorder or negative transformation:

*‘Institutions tell them (adolescents) about their rights… and that’s where the problem lies’ (Teacher, male)*.

This discrepancy suggests that, while adolescents experience these challenges as barriers to learning, teachers view them through the lens of discipline and control.

#### 3.1.2 Emotional and relational challenges

Emotional and relational challenges are linked to processes of self-regulation, managing meaningful relationships, and protecting emotional integrity. Adolescents described constant efforts to “be okay” or “be strong” in the face of interpersonal conflicts.

One adolescent noted:

*‘The biggest challenge was to be strong and not let others hurt me’ (Adolescent, female)*.

Difficulties with self-control were also identified:

*‘Sometimes I cannot control myself’ (Adolescent, female)*.

Female adolescents tended to describe experiences related to resentment, overthinking, and sadness in greater depth. In contrast, males often placed the challenge on the behaviour of others or on external events, with less explicit emotional elaboration.

In contrast, teachers enrich this category by situating emotional and relational challenges within the group dynamics characteristic of adolescence, linked to coexistence, constant interaction among peers, and the interpersonal conflicts that arise in these settings. From this perspective, emotional tensions are not interpreted solely as individual processes of self-regulation, but as relational phenomena that are expressed in group dynamics:

*‘Adolescents are mostly in groups… sometimes they argue, that’s part of being adolescents’ (Teacher, female)*.

This perspective offers a more contextual understanding of emotional challenges by emphasising their interactive and situational nature, in contrast to the subjective experiences reported by the adolescents themselves.

#### 3.1.3 Economic challenges

Material deprivation was described as a persistent experience that shapes opportunities, priorities and future prospects. Economic constraints are perceived not only as a lack of goods, but also as a structural factor that influences everyday experiences.

During a focus group, one adolescent noted:

*‘We do not have enough money to buy something we need’ (Adolescent, male)*.

The adolescents also identified finances as a barrier to accessing professional help and/or specialised services:

*‘Finances can prevent you from getting professional help’ (Adolescent, female)*.

In some cases, this situation meant taking on work responsibilities at an early age:

*‘I got up at 5 a.m. to help my mum […] then I went to clean a hotel’ (Adolescent, female)*.

Work coexisted with study, leading to fatigue and a reorganisation of academic priorities.

From the teachers’ perspective, there is a clear consensus that financial precariousness is a structural constraint that affects academic performance and determines future educational prospects:

*‘There’s no financial support at home… and that affects their studies’ (Teacher, male)*.

Teachers also point out that some adolescents work to cover personal costs, such as mobile data, which creates new pressures on their use of time and daily routines:

*‘They work to earn money… and spend it on data or games’ (Teacher, male)*.

This contrast reveals that, while adolescents emphasise the direct experience of deprivation and the struggle with material aspects at a given moment, teachers broaden the understanding by linking economic precariousness to uncertain future educational trajectories and to digital consumption patterns which, in some cases, may reinforce forms of escapism from the situation.

#### 3.1.4 Daily challenges

Daily challenges included time management, domestic responsibilities, unexpected situations, and physical discomfort. These experiences reflect tensions between obligations and personal desires.

*‘The biggest challenge was organising my time’ (Adolescent, female)*.

Experiences of insecurity in the face of unexpected interactions with strangers also emerged:

*‘I was scared […] I didn't know what to do’ (Adolescent, female)*.

In addition, difficulties related to sleep, eating, and physical ailments were mentioned, which affect daily performance.

Although teachers do not focus directly on the everyday events mentioned by adolescents, they interpret them within a broader framework associated with disorganisation, a lack of routine and difficulties with self-regulation, linked to limited supervision:

*‘They come in sleepy… they’ve been playing all night’ (Teacher, male)*.

This difference reflects the fact that, while adolescents describe specific day to day experiences, teachers reframe them as indicators of habits and discipline, introducing a more normative interpretation of everyday functioning within the school environment.

#### 3.1.5 Family challenges

Family challenges were associated with conflicts, misunderstanding, and a perception of poor emotional support. Arguments with the mother and difficulty communicating with the father were recurring themes.

*‘I always fight with my mum’ (Adolescent, female)*.

*‘My dad doesn't understand what I want’ (Adolescent, female)*.

In the focus groups, stories emerged of psychological violence and parental arguments that have an emotional impact on children:

*‘Parents argue and children listen […] that affects psychologically’ (Adolescent, female)*.

Among male adolescents, there was a tendency to normalise situations of violence or parental criticism:

*‘If my dad told me I was behaving badly, it would be okay for him to hate me’ (Adolescent, male)*.

Overall, the challenges perceived by adolescents reflect individual experiences, as well as the interaction between structural conditions, such as economic and family circumstances, school dynamics, and subjective processes of emotional regulation. These experiences constitute the scenario in which their general resources and their capacity for agency are mobilised or inhibited.

From the teachers’ perspective, there is a consensus on the high prevalence of intrafamily conflicts and difficulties in communicating with parents as risk factors. However, teachers broaden this interpretation by incorporating the influence of the digital environment, particularly the internet and social media, as a factor that interferes with family dynamics and the development of cultural references, and that are not appropriate for them:

*“They have communication problems with their parents… and follow models from the internet” (Teacher, male)*.

This contrast highlights that, while adolescents describe conflicts from their direct emotional experience, teachers situate them within a broader framework that combines family dynamics and external sociocultural factors, offering a more structural interpretation of the problem.

### 3.2 General resistance resources identified

Adolescents identify and mobilise various general resistance resources (GRR) in response to the challenging situations mentioned above. These resources encompass personal, relational, digital and institutional dimensions, and serve the functions of emotional regulation, daily support and active coping. However, barriers to their recognition and activation are also evident. Supplementary Figure 2 shows the most common salutogenic resources.

A comparative analysis reveals a partial convergence between the perspectives of adolescents and teachers. While adolescents identify and mobilise a wide range of resources, including personal, family, social, digital and institutional dimensions, teachers tend to perceive these resources as more limited and concentrated primarily within the family and informal networks of trust. They point to a limited seeking of professional help and a greater reliance on close relationships. This contrast suggests that, although adolescents recognise multiple sources of support, their effective mobilisation may be influenced by accessibility, trust and immediate relational dynamics. Although teachers tend to adopt a more restricted and risk-oriented view, some elements of alignment can be identified, particularly in the recognition of close relationships and support networks as relevant resources. However, these are often interpreted by teachers in terms of need and dependency, whereas adolescents frame them as active sources of support and agency.

#### 3.2.1 Personal resources

Personal resources include emotional, cognitive, physical and spiritual abilities that can be mobilised without necessarily relying on external support. In terms of attitude, self-confidence, self-motivation, calmness and assertive communication emerge as tools for dealing with interpersonal and academic conflicts.

In the cognitive dimension, “thinking before acting,” reinterpreting the situation or prioritising tasks allows a sense of control to be regained. Physically, sleeping, playing sports or doing breathing exercises function as self-regulation mechanisms. Spiritually, belief in God is a source of strength, hope and meaning.

From a gender perspective, men tend to emphasise action-oriented and distraction strategies, while women highlight expressive and spiritual resources linked to emotional containment.

*‘Praying to God helps me’ (Adolescent, female)*.

*‘My mindset helps me understand things better’ (Adolescent, male)*.

In contrast, teachers do not recognise adolescents’ internal resources, but rather view them as aspects still in development that depend on external guidance. From this perspective, these resources are interpreted as aspects that require guidance, support and adult experience in order to be properly recognised and activated. However, some teachers point out that they identify and rely on faith-based organisations as a legitimate community resource for strengthening support for adolescents.

*‘They always need guidance from an adult’ (Teacher, female)*.

This contrast suggests that, while adolescents perceive and utilise personal resources, teachers interpret them as capacities still under development that require adult support.

#### 3.2.2 Family and social resources

The family is the primary support network. The mother appears to be the central figure for listening and emotional support, while siblings provide academic support and advice. Peers serve as a source of relief and daily companionship.

Gender differences can be observed: male adolescents associate family support mainly with economic aspects or serious situations, while female adolescents emphasise frequent conversation and emotional support.

*‘I talk to my mum and my best friend’ (Adolescent, female)*.

*‘My mum gives me advice’ (Adolescent, male)*.

From the teachers’ perspective, there is a consensus on recognising the family as the central source of support. However, they broaden this view by pointing out that adolescents’ resources are centred on close, trusting relationships, including their partner as a significant source of emotional support:

*‘My student said to me: He’s the only person who understands me (partner)’ (Teacher, female)*.

Likewise, teachers highlight that many adolescents come from complex family backgrounds, which increases the need for external guidance:

*‘Some come from dysfunctional families… they need guidance’ (Teacher, female)*.

This contrast shows that, although both agree on the centrality of close relationships, teachers interpret them in terms of need and vulnerability, while adolescents experience them as active sources of support. This suggests that, despite differing interpretations, there is a shared recognition of relational resources as central, indicating a potential point of convergence between adolescent and adult perspectives.

#### 3.2.3 Digital resources

Mobile phones are emerging as central, multifunctional devices. They are used to search for academic and health information, communicate with support networks, and regulate emotions through music, social media, or video games. Artificial intelligence tools are used for immediate academic support, such as chat gpt.

Male adolescents emphasise instrumental use and digital gaming, while female adolescents prioritise seeking guidance and informational support.

*‘I searched the internet and found a delicious recipe’ (Adolescent, female)*.

*‘When I need information about my health, or to distract myself, I use AI and ChatGPT*.*’ (Adolescent, male)*.

In contrast, while teachers recognize that adolescents make extensive use of technology, they tend to view it in a predominantly negative light, seeing it more as a means of escapism than as a resource. From their perspective, the intensive use of social media and online games is interpreted as a form of refuge from a lack of attention or affection within the family environment, which can affect concentration and motivation towards academic activities:

*‘They get caught up in games… and studying becomes difficult’ (Teacher, male)*.

*“They take refuge in virtual games more than anything else” (Teacher, male)*.

This contrast suggests that, while adolescents perceive technology as a tool for support and regulation, teachers interpret it primarily as a factor of distraction and deregulation, highlighting a difference in the assessment and interpretation of digital resources.

#### 3.2.4 Institutional and community resources

The school, teachers and school psychologist are recognised as sources of academic and emotional support. Health centres and protection services are also mentioned as formal resources in serious situations, although access to them often depends on adult mediation. Particularly in situations of discrimination, violence, or when some family members did not take them seriously.

*‘We would talk to a teacher we trust*.*’ (Adolescent, female)*.

*‘We could write or take up a hobby, or even go to a psychologist’ (Adolescent, female)*.

*‘I report it to the judicial authorities, the police’ (Adolescent, male)*.

Community and natural spaces serve as settings for emotional regulation and well-being.

*‘My community is kind and supportive, which makes me feel safe and calm’ (Adolescent, female)*.

From the teachers’ perspective, there is a consensus that students rarely seek formal help, despite the availability of institutional resources. Teachers indicate that adolescents tend to focus their support strategies on spaces of immediate trust, such as their partner or other close relationships, rather than turning to adult figures or professional services:

*‘Very few adolescents approach a teacher or tutor’ (Teacher, female)*.

Furthermore, teachers do not explicitly recognise community or natural settings as relevant resources, and, in some cases, attribute a potentially problematic role to certain institutions, considering that they promote a form of ‘empowerment’ perceived as negative:

*‘Institutions or non-governmental organisations talk about their rights… and that’s where the problem starts’(Teacher, male)*.

In contrast, adolescents do identify a wider range of resources, including institutional and community spaces and everyday environments, although their access to these is often mediated by adults or by the level of trust. This difference shows that, although formal and community resources are recognised by adolescents, teachers tend to favour a more restricted view, centred on immediate relationships and a critical interpretation of the role of institutions. However, this perspective also reflects that teachers recognise the importance of close and accessible support structures, particularly those based on trust and proximity, even if they tend to frame them in terms of supervision and guidance rather than as expressions of adolescent agency.

#### 3.2.5 Difficulties in recognising resources

Despite the wide identification of resources, some adolescents have difficulty naming or activating them in contexts of distress, expressing feelings of loneliness or helplessness, or simply ignorance. This gap is more common in male adolescents, who show less emotional expression and greater ambiguity in identifying support.

*‘I did not know who to turn to when I was being discriminated’ (Adolescent, female)*.

In line with what has been described in the previous points, from the teachers’ perspective this limitation is interpreted not merely as a situational difficulty, but as a more structural characteristic linked to dependence on adult figures and to the limited autonomy in mobilising resources that is typical of adolescence. Teachers tend to consider that adolescents do not fully possess their own resources, but rather require constant guidance to identify and utilise them.

This contrast highlights that, while adolescents recognise and can articulate the existence of resources, even if they do not always succeed in activating them, teachers tend to emphasise their insufficiency and dependence on external support, reinforcing an interpretation that prioritises deficits over emerging capabilities.

### 3.3 Conditional agency and coping strategies

Adolescents described different ways of responding to everyday challenges, reflecting varying degrees of agency shaped by personal reflection, available support, and contextual constraints. These responses ranged from deliberate and problem-oriented actions to more avoidant or emotionally driven coping strategies. Supplementary Figure 3 shows the most common responses.

A comparative analysis reveals a difference in the interpretation of this agency: while adolescents describe it as a dynamic capacity that is context-dependent and present in them, teachers tend to perceive it as incomplete, still developing and heavily reliant on adult supervision, more often emphasising its limitations than its potential. However, this perspective also involves a partial recognition of adolescents’ capacity for action, as teachers do acknowledge that such a capacity exists.

#### 3.3.1 Capacity for agency

Adolescents not only identify challenges, but also mobilise resources to address them, demonstrating a dynamic and contextual capacity for agency. Agency does not emerge as a stable trait, but rather as a capacity that is activated, modulated or restricted according to the perceived severity of the challenge, its personal impact and the availability of resources.

Two predominant forms are identified. The first corresponds to active personal agency, where adolescents take direct responsibility for problem solving: they reflect, regulate emotions, practise self-care, seek information, solve tasks autonomously and strategically request support when they deem it necessary. In this sense, seeking help does not imply dependence, but rather the deliberate exercise of autonomy.

The second corresponds to conditioned agency, in which action depends on contextual factors, especially the perceived impact of the challenge and the resources required to address it. In these cases, resolution usually follows a sequence: an initial individual attempt followed by recourse to third parties if the situation exceeds their perceived capabilities. When the challenge is perceived as manageable at an individual level, action tends to be autonomous; however, when it involves greater emotional or contextual complexity, it may be postponed or require external support.

From a gender perspective, male adolescents emphasise material autonomy and practical resolution, while female adolescents link agency to process. Adolescents themselves distinguish between challenges they can handle independently and those that require external support:

*‘I usually do it myself (solve the problem)’ (Adolescent, male)*.

*‘Depending on the severity of the problem*. *If it’s simple, I solve it myself’ (Adolescent, female)*.

Although teachers recognize the existence of agency, they describe it as limited and dependent on adult guidance, noting that autonomous decision-making is still in the process of development:

*‘They (adolescents) will always wait for a response first’ (Teacher, female)*.

This difference highlights a gap between the agency experienced by adolescents and the agency perceived by adults, where the former is constructed as a situated practice and the latter as a capacity that is still incomplete.

#### 3.3.2 Coping strategies

The coping strategies identified are diverse and dynamic, and are linked to the resources and agency described above.

Active strategies are observed, aimed at solving the problem or reducing its impact: dialogue, verbal confrontation, academic planning, reporting injustices, seeking professional guidance, and making practical decisions. Coping often begins autonomously before seeking external support, regardless of the results.

Avoidance strategies are also evident, focusing on regulating discomfort rather than resolution: ignoring the problem, isolating oneself, taking refuge in technology, sleeping, or suppressing emotions. In these cases, the action is aimed at immediate relief.

Impulsive responses are also identified, particularly in interpersonal conflicts, where emotional reaction precedes reflection. Less frequently, maladaptive strategies or deliberate inaction appear, such as self-harm, associated with uncertainty, normalisation of violence, or perception of a lack of alternatives.

Barriers to coping include cognitive factors (confusion, overthinking), emotional factors (fear, pride), relational factors (lack of trust), and structural factors (economic limitations, age).

Successful coping strengthens self-efficacy and a sense of personal growth; unsuccessful coping generates learning or awareness of limits.

From a gender perspective, men tend to emphasise instrumental or confrontational coping, while women favour reflective and relational strategies.

*‘I faced and calmed down, thanks to my friends*.*’(Adolescent, female)*.

*‘I listen to music, play football, chat online, etc*.*’ (Adolescent, male)*.

From the teachers’ perspective, they tend to highlight avoidance or reactive strategies as predominant, particularly in vulnerable situations. They point to the heavy use of technology, early entry into the workforce, or escapism as common coping mechanisms:

*‘They escape through games, work or alcohol*.*’ (Teacher, male)*.

They also highlight higher-risk strategies that do not always emerge in adolescents’ accounts, such as self-harm, because they feel the pain goes away:

*‘Many engage in cutting to cope’ (Teacher, female)*.

This contrast suggests that, while adolescents report a combination of active and regulatory strategies, teachers tend to interpret coping from a perspective centred on risk and avoidance, broadening our understanding of the phenomenon by incorporating practices that are less explicitly mentioned.

## 4 Discussion

The results of this study show that the experiences of adolescents in vulnerable contexts are shaped by the interplay of relational factors (family, peers, school), structural dimensions (economic constraints) and subjective personal dimensions, such as emotional regulation and identity. However, beyond identifying challenges and resources, the findings provide an understanding of how these interact dynamically in the activation of general resistance resources (GRR) and in the construction of their sense of coherence (SOC). In this process, agency emerges as a contextualised capacity, modulated both by structural conditions and by social interpretations, including perceptions of the adult environment, which influence coping strategies for perceived challenges.

These results are consistent with the ecological model of human development (42), and The Lancet Commission on Adolescent Health (43), which highlight the role of structural determinants in well-being. However, this study expands on these perspectives by demonstrating how these determinants not only generate challenges, but also condition the activation of resources, the perception of control and the possibilities.

### 4.1 Dynamics of GRRs and the dimensions of SOC

From the perspective of salutogenesis theory, the identified resources, personal, family, social, digital and institutional, can be understood as GRRs that contribute to the construction of SOC (18, 20, 44). However, the results show that these resources do not operate in isolation, but are articulated in distinct ways across the three dimensions of SOC: comprehensibility, manageability and meaning.

In terms of comprehensibility, adolescents use personal and relational resources to make sense of their experiences, particularly in academic and family contexts. Strategies such as “thinking before acting”, spirituality, talking to peers or using digital tools enable them to manage situations initially perceived as difficult. However, the divergence from the teachers’ perspective, which interprets these same behaviours as a lack of boundaries or disorganisation, suggests that adolescents’ construction of comprehensibility is not always recognised or validated, specially in the school environment.

In terms of manageability, family (particularly maternal support), social and institutional GRRs emerge as key resources that enable challenges to be perceived as manageable. For example, adolescents describe going to their mothers or trusted persons to talk and seek guidance, which facilitates emotional regulation and decision-making. Likewise, strategies such as seeking academic help, using digital tools or approaching teachers in specific situations reinforce the perception that support is available. Spirituality also emerges as a significant resource, as it enables situations of high uncertainty to be transformed into emotionally manageable experiences through faith. However, manageability is constrained by structural factors, such as economic difficulties or dependence on adults, which limit effective access to these resources and force individuals to prioritise immediate responses over sustainable solutions.

Spiritual and community resources appear to be influenced in part by the cultural context of the region. With regard to spiritual resources, their presence is linked to the predominance of Catholicism (45), as well as to the influence of family and institutional practices, as noted by adolescents and teachers. However, these resources emerge primarily as forms of subjective emotional support, rather than as formally structured religious practices.

Community resources, meanwhile, are associated with the availability and significance of natural spaces, which adolescents use as settings for emotional regulation and refuge, in line with the geographical characteristics of the environment (45). Furthermore, the condition of structural vulnerability favours the presence of programmes promoted by external organisations, which could explain why teachers’ discourse perceives certain institutions as promoters of rights that, on occasion, come into conflict with local norms of authority. Together, these findings suggest that the resources identified do not respond to homogeneous cultural patterns, but rather to situated responses, shaped by the opportunities and limitations of Acobamba’s social and geographical environment.

Regarding digital tools, an emerging finding of particular contemporary relevance is the incorporation of generative artificial intelligence, specifically ChatGPT, as a digital GRR in contexts of rural vulnerability. Adolescents strategically utilise it to obtain immediate academic support, compensating for limitations within the school or family environment arising from gaps in teaching or support. This use of AI can be understood as a form of proactive agency, through which adolescents activate available resources to tackle academic challenges and reduce structural inequalities. In this sense, tools such as ChatGPT function as a form of cognitive support, facilitating access to information, the comprehension of difficult to understand content, and the development of problem-solving skills. Recent evidence suggests that the use of AI is associated with improvements in academic performance, motivation and autonomy in learning, particularly in resource-limited contexts (46, 47).

In terms of meaning, it is identified as the motivational factor that promotes the mobilization of resources. Adolescents give meaning to their experiences through elements such as personal goals, meaningful relationships and the desire for self-improvement. For example, facing situations that involve temporary separation from loved ones, such as attending school despite the desire to stay at home, is interpreted as a necessary effort with a long-term purpose. Similarly, taking on responsibilities, such as studying despite financial difficulties or balancing study and work, takes on a meaning linked to improving one’s own and one’s family’s living conditions. This component allows everyday challenges to be interpreted not merely as obstacles, but as part of a purposeful process, which is key to sustaining effort in adverse contexts.

The results of this study broaden the framework by showing that the mere existence of resources does not guarantee their activation, as reflective and contextual capacity is necessary to recognise and use them, a process closely linked to agency.

### 4.2 Bounded agency and SOC in contexts of vulnerability

The agency is presented as a dynamic and situated capacity. Adolescents describe deliberate actions such as reflecting, dialoguing, regulating emotions, seeking information or support, which position them as active agents in the construction of their well-being. However, this agency appears to be conditioned by the severity of the challenge, the availability of support, and structural constraints, which coincides with the notion of “bounded agency,” where the capacity to act is exercised within margins defined by the social and economic context (48, 49). In contexts of material scarcity, agency tends to be activated primarily in response to economic needs or critical situations, whereas in emotional conflicts it may be restricted or postponed.

Although the concept of agency may seem similar to that of resilience, it is important to distinguish between them. Resilience refers to the ability to adapt in the face of adversity (50, 51), and has traditionally been approached from a pathogenic perspective centred on the response to harm (58). In contrast, conditioned agency emphasises the capacity to act and make decisions within structurally limited contexts, understood as adolescents’ ability to influence their environment, make decisions and mobilise resources in line with their goals and contexts (52). This means it describes not only how adolescents respond to challenges, but also how they actively negotiate them within the available constraints.

In contexts of economic insecurity, agency is proactively channelled into survival strategies, such as taking on early work to cover their own or their family’s needs, thereby reinforcing manageability in functional terms. However, in the emotional and relational sphere, this same agency can take on avoidant or reactive forms when resources are perceived as insufficient. For example, adolescents describe resorting to isolation, intensive mobile phone use or distracting activities such as listening to music or playing online games to cope with loneliness or emotional distress, prioritising immediate relief over problem-solving.

In this context, manageability is not always built on resolution-oriented coping strategies, but also through responses focused on immediate emotional regulation, such as avoiding the problem, distracting oneself or postponing action, which enable individuals to maintain their day-to-day functioning in adverse circumstances. However, these strategies may limit long-term well-being, as they do not address the underlying causes of the challenges (for example, family conflicts or persistent emotional difficulties), and in some cases may even lead to higher-risk responses identified by teachers, such as impulsive or self-harming behaviours.

Furthermore, the contrast with the teachers’ perspective reveals a gap in the interpretation of agency: while adolescents perceive themselves as active agents who make decisions based on their available resources, even if these are not always evident, teachers tend to conceptualise these responses as evasive or insufficient. This discrepancy highlights that adolescent agency is not only conditioned by context, but also by the interpretative frameworks through which it is assessed, which can influence its recognition and reinforcement.

The gender differences observed reinforce international evidence on differences in coping. Female adolescents tend to use reflective and relational strategies, while male adolescents favour instrumental or avoidant responses, with less emotional verbalisation. Recent studies have documented similar patterns, noting that gender differences in coping influence help-seeking and emotional regulation (53). These findings suggest the need for gender-sensitive interventions that strengthen emotional literacy in male adolescents and promote leadership opportunities and structural autonomy in female adolescents.

The coping strategies identified (active, avoidant, impulsive and, to a lesser extent, maladaptive) should not be understood exclusively as functional or dysfunctional, but rather as adaptive responses in specific contexts and situations. Research on adolescent coping suggests that avoidance can serve a regulatory function in environments perceived as uncontrollable (54). However, when not accompanied by structural or relational support, it can perpetuate discomfort.

### 4.3 Interpretative tension between the asset-based perspective and the deficit approach

A key finding of this study is the existence of an interpretative tension between the way in which adolescents identify and mobilise their resources and the way in which these are understood by teachers. While adolescents recognise and activate various GRRs, such as personal, peer support or the use of digital technologies, as valid strategies for coping with their challenges, teachers tend to interpret these same elements from a deficit or risk focused perspective. This tension reflects what the literature has termed adult-centred or deficit approaches to adolescence (55), which can limit and skew perceptions about them.

For example, adolescents describe mobile phone use as a tool for finding information, managing emotions or maintaining social connections; however, teachers view it primarily as a form of evasion that affects concentration and academic performance, and do not regard it as a resource. Similarly, support from a partner or close friends is experienced by adolescents as a significant emotional resource, while teachers associate it with dependency or potentially risky decisions, linked to a waste of time that interferes with academic performance. In addition, adolescents identify institutional resources, such as school staff, protection services or community institutions, as potential sources of support in situations of conflict or vulnerability. In contrast, some teachers perceive these same institutions as problematic, arguing that they may promote a form of “empowerment” that encourages attitudes or expectations misaligned with the adolescents’ social context, thereby framing them more as sources of risk than as legitimate resources.

This difference is not merely a matter of perception; it has direct implications for the recognition of resources. When the resources identified by adolescents are not recognised and supported, or are negatively reinterpreted by adults, their potential contribution to strengthening the SOC is limited (56). In this context, this difference in perspectives can hinder the effective mobilisation of resources and subsequent capacity for agency and coping, by generating persistent stress and a lack of support (57).

However, this tension does not imply a complete absence of recognition from the adult perspective. Teachers do acknowledge that adolescents rely on close relationships and are capable of acting in response to challenges, although this agency is often framed as limited and requiring guidance. This suggests that the divergence lies not only in the identification of resources, but in how these are interpreted, valued, and legitimised within the adult framework.

### 4.4 Contributions to salutogenic theory and the expansion of the conceptual framework

The findings of this study are consistent with salutogenic theory and also extend it in contexts of vulnerability. Firstly, they confirm that adolescents not only possess GRRs, but also identify, interpret and mobilise them dynamically depending on the context and nature of the challenge, which reinforces the processual nature of the SOC. For example, a single resource, such as the use of a mobile phone, can function as academic support or emotional regulation, or as an avoidance strategy, depending on the situation. This demonstrates that GRRs are neither static nor inherently positive, but that their function depends on their contextual activation.

Furthermore, the findings expand our understanding of SOC by showing that its development depends not only on the availability of resources, but also on their recognition and validation within the social environment. The difference between how adolescents identify resources and how teachers interpret them suggests that the institutional environment can either facilitate or limit the consolidation of SOC. This finding introduces the role of social validation as a key element in the effectiveness of GRRs, in line with approaches that highlight the relational nature of salutogenesis.

This study evidences the importance of incorporating multiple perspectives into the salutogenic analysis. Bringing together the voices of adolescents and teachers made it possible to identify not only resources and capabilities, but also interpretative tensions and risks that do not emerge in a uniform manner. For example, while adolescents highlight relational and emotional coping strategies, teachers draw attention to risk behaviours that are less explicitly mentioned. This pluralistic approach lends rigour and depth to the analysis of GRRs and agency.

The findings show that, in contexts of vulnerability, resources and agency are shaped by survival dynamics. In these scenarios, the mobilisation of resources may be directed towards meeting immediate needs, such as working or avoiding emotional distress, rather than promoting sustained well-being, thereby broadening our understanding of the SOC as a process that is also adaptive to adverse conditions. This also opens up a significant line of research into how to move from survival-focused coping strategies towards processes that promote sustained well-being over time.

Finally, this study incorporates a gender perspective as a relevant contribution to salutogenic theory. The differences observed indicate that female adolescents tend to mobilise relational and reflective resources, while male adolescents favour more instrumental or avoidant strategies. This suggests that the construction of SOC and the activation of GRR are mediated by gender norms and socialisation, reinforcing the need for salutogenic approaches that are sensitive to these differences.

Overall, the results contribute to a more contextualised and critical understanding of salutogenesis, highlighting the importance of integrating individual capabilities, social validation and structural conditions into the analysis of adolescent well-being.

From a public health perspective, the findings suggest a need for context-specific school-based interventions that include a resource mapping exercise designed jointly by adolescents and teachers, which would help students identify and mobilise their own GRRs and strengthen their capacity for action. It is also essential to collaborate with teachers to promote a change in perceptions of adolescence, promoting a salutogenic approach that values students’ resources and capabilities, as well as encouraging the joint design of interventions with various stakeholders in the educational sector to improve their relevance and effectiveness. Finally, digital literacy focused on health promotion would enable the use of technologies as support resources for adolescents and pedagogical strategies for teachers. Together, these actions strengthen adolescents’ capacity for action and help transform the school environment into a more conducive setting that promotes health, in line with a more contextualised and critical understanding of salutogenesis that integrates individual capabilities, social validation and structural conditions.

### 4.5 Limitations

This study has limitations. It was conducted in a single educational institution, which restricts the generalisation of results. The qualitative design does not allow for the measurement of objective SOC levels or the establishment of causal relationships. However, it provides a deep contextual understanding of how adolescents in a vulnerable environment shape their well-being from a salutogenic perspective.

Although this study examines gender differences in depth, other dimensions of intersectionality, such as family structure, religious identity or socioeconomic status, did not show significant variation within the sample. The participants shared relatively homogeneous sociodemographic characteristics, including similar family living patterns, a predominance of Catholic religious affiliation, and conditions of economic vulnerability typical of the regional context, which was consistent in both the adolescents’ accounts and the teachers’ perceptions.

This homogeneity limited the possibility of comparatively exploring the weight of these factors in experiences and coping strategies. In this context, the findings suggest that the identification of resources and the observed ‘bounded agency’ respond, to a large extent, to shared structural conditions, where socio-economic vulnerability operates as a cross-cutting determinant that shapes both the challenges and the available resources.

However, it is important to note that these dimensions were not explored in depth through specific questions; therefore, future research should incorporate a more explicit intersectional approach that allows for an analysis of how these variables interact in shaping adolescent well-being and agency.

In addition, the sample presented a gender imbalance, with a higher proportion of female participants, as participation was voluntary and based on adolescents’ willingness to take part. This may have limited the depth and diversity of male perspectives, particularly in relation to emotional expression, coping strategies, and resource identification.

Overall, the results support a systemic interpretation of adolescent well-being. Adolescents are not passive recipients of risk, but actors who negotiate, interpret, and respond to complex structural conditions. Strengthening their ability to recognise and mobilise resources, coupled with structural transformation, is a promising avenue for health promotion in vulnerable contexts.

## 5 Conclusion

This study demonstrates that well-being among adolescents in vulnerable contexts is a dynamic process that emerges from the interaction between structural conditions, relational contexts and subjective capacities. This process is mediated through the activation of GRRs, which contribute to the construction of the SOC in its dimensions of comprehensibility, manageability and meaning.

The results show that the mere availability of resources is not enough if adolescents are unable to recognise them, put them into practice and make sense of them. In this context, SOC is not constructed solely through access to resources, but also through their situated use and their validation within the social environment. Furthermore, it is confirmed that adolescent agency is dynamic but bounded, as its exercise is conditioned by structural constraints that limit real opportunities for action.

A significant contribution is the identification of a tension between the way in which adolescents mobilise their resources and how these are interpreted by the adult environment. Adolescents recognise diverse assets, including digital, relational and personal resources, while teachers recognise adolescents’ social resources and emerging capacity for agency, although they tend to interpret these from a perspective that focuses on their deficiencies and risks. This difference may limit the legitimisation of resources and affect the development of SOC, particularly in school contexts.

Furthermore, the findings extend salutogenic theory by showing that, in contexts of vulnerability, the mobilisation of resources may be directed towards meeting immediate needs rather than promoting sustained well-being. This finding highlights the need to explore further how to move from survival-focused coping strategies towards processes that foster long-term pathways to well-being.

Finally, this study underscores the need to move towards more comprehensive and context-specific health promotion approaches. It is not enough to strengthen individual capacities; it is necessary to transform the structural conditions and institutional dynamics that limit adolescent agency. In this regard, there is a need to promote non-adult-centred models that recognise, validate and enhance adolescents’ resources and capacities, while also incorporating gender and context sensitive perspectives to promote more equitable and effective interventions.

## Data Availability

The data generated and analysed during the study are not publicly available due to ethical and confidentiality restrictions related to the participation of adolescents, but the corresponding author can provide them upon reasonable request and with the appropriate institutional authorisation. Requests to access the datasets should be directed to u1997507@campus.udg.edu.
