# Supplementary material for: Challenges, salutogenic resources and adolescent agency: a qualitative study in a public school in Peru
Source: Front Public Health. 2026 Apr 29;14:1824105. doi: 10.3389/fpubh.2026.1824105 (PMC13170679; doi:10.3389/fpubh.2026.1824105)
Supplement: Supplementary file 1 [file Data_Sheet_1.docx]

***Supplementary Material***

## Supplementary Tables

**Table 1. Coding Structure:** From initial codes to core themes with representative quotes

| *Initial code* | *Subtheme* | *Theme* | *Example quote* |
| --- | --- | --- | --- |
| *Difficulty understanding, concentrating in class, and avoiding distraction* | *Distraction as a barrier to learning* | *Academic challenges* | *“The most difficult thing for me is understanding what the teacher explained, because I was distracted and felt embarrassed” (Adolescent, female, personal diary)* |
| *Fulfilling responsibilities vs. desire to do other things* | *Tension between duties and personal desires* | *Everyday challenges* | *“The most difficult thing was leaving home (to go to school), because I didn’t want to be away from my sister…” (Adolescent, female, personal diary)* |
| *Trying to change mood or “feel okay”* | *Emotional self-regulation as a challenge* | *Emotional and relational challenges* | *“The most difficult thing was trying to feel okay, because I thought nothing would make me laugh” (Adolescent, female, personal diary)* |
| *Seeking meaning through religion* | *Spirituality as a resource* | *Spiritual resources* | *“Asking God helps me with the difficulties I have” (Adolescent, female, personal diary)* |
| *Talking to trusted people to relieve confusion* | *Seeking interpersonal support* | *Family and/or social resources* | *“Talking to someone I trust helps me when I feel confused” (Adolescent, male, personal diary)* |
| *Taking action independently in response to challenges* | *Individual active coping* | *Personal resources* | *“When I feel sad… I try to solve my own problems” (Adolescent, male, personal diary)* |
| *Seeking informal work through family networks* | *Informal economic strategies mediated by family* | *Conditioned agency* | *“They wouldn’t give a job to a minor, but he could help his aunt…” (Focus group, males)* |
| *Using a mobile phone to cope with loneliness* | *Mobile phone use as a coping strategy* | *Active coping with challenges* | *“When we feel lonely… we talk on the phone and distract ourselves” (Focus group, females)* |
| *Adolescent empowerment perceived negatively* | *Empowerment as a threat from teachers’ perspective* | *Challenges perceived by teachers* | *“They feel empowered… and there is no longer respect for teachers…” (Tutor, female)* |
| *Perceived rebellious behaviour* | *Adult representations of adolescence* | *Perceptions of adolescents* | *“What young people do is rebel…” (Tutor, male)* |

## Supplementary Figures
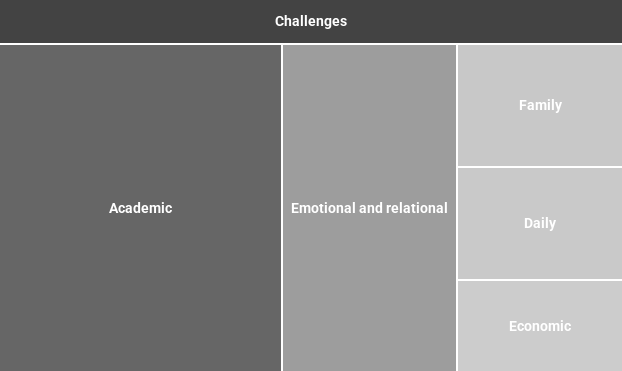


**Supplementary Figure 1.** Distribution of perceived adolescent challenges. The area of each rectangle represents the relative frequency with each category.

**
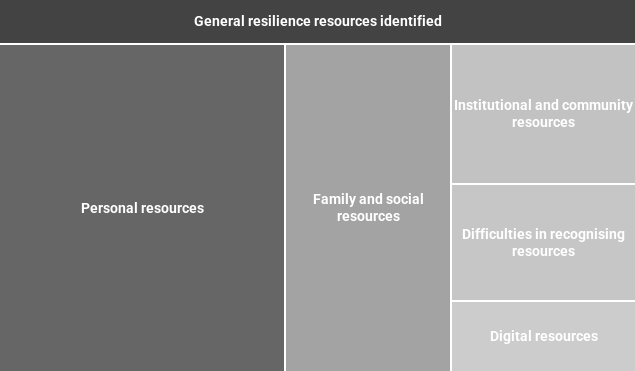
**

**Supplementary Figure 2**. Distribution of salutogenic resources identified by adolescents. The area of each rectangle represents the relative frequency with each category.

**
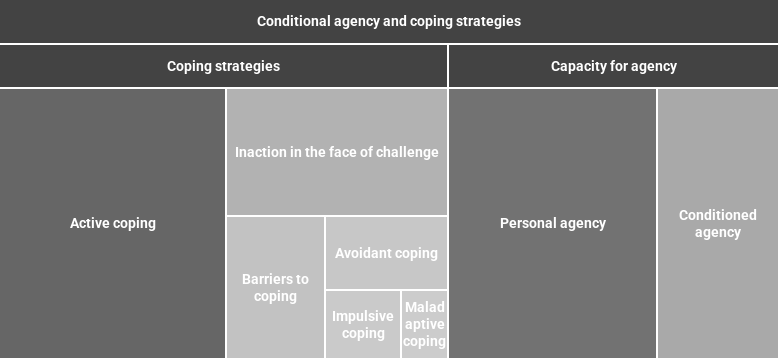
**

**Supplementary Figure 3**. Distribution of agency and coping strategies by adolescents. The area of each rectangle represents the relative frequency with each category.
